# Molecular Mechanisms of *Staphylococcus* and *Pseudomonas* Interactions in Cystic Fibrosis

**DOI:** 10.3389/fcimb.2021.824042

**Published:** 2022-01-06

**Authors:** Lalitha Biswas, Friedrich Götz

**Affiliations:** ^1^ Centre for Nanosciences and Molecular Medicine, Amrita Vishwa Vidyapeetham, Kochi, India; ^2^ Microbial Genetics, Interfaculty Institute of Microbiology and Infection Medicine Tübingen (IMIT), University of Tübingen, Tübingen, Germany

**Keywords:** cystic fibrosis, Staphylococcus aureus, Pseudomonas aeruginosa, antagonism, adaptation, co-existence

## Abstract

Cystic fibrosis (CF) is an autosomal recessive genetic disorder that is characterized by recurrent and chronic infections of the lung predominantly by the opportunistic pathogens, Gram-positive *Staphylococcus aureus* and Gram-negative *Pseudomonas aeruginosa.* While *S. aureus* is the main colonizing bacteria of the CF lungs during infancy and early childhood, its incidence declines thereafter and infections by *P. aeruginosa* become more prominent with increasing age. The competitive and cooperative interactions exhibited by these two pathogens influence their survival, antibiotic susceptibility, persistence and, consequently the disease progression. For instance, *P. aeruginosa* secretes small respiratory inhibitors like hydrogen cyanide, pyocyanin and quinoline *N*-oxides that block the electron transport pathway and suppress the growth of *S. aureus*. However, *S. aureus* survives this respiratory attack by adapting to respiration-defective small colony variant (SCV) phenotype. SCVs cause persistent and recurrent infections and are also resistant to antibiotics, especially aminoglycosides, antifolate antibiotics, and to host antimicrobial peptides such as LL-37, human β-defensin (HBD) 2 and HBD3; and lactoferricin B. The interaction between *P. aeruginosa* and *S. aureus* is multifaceted. In mucoid *P. aeruginosa* strains, siderophores and rhamnolipids are downregulated thus enhancing the survival of *S. aureus*. Conversely, protein A from *S. aureus* inhibits *P. aeruginosa* biofilm formation while protecting both *P. aeruginosa* and *S. aureus* from phagocytosis by neutrophils. This review attempts to summarize the current understanding of the molecular mechanisms that drive the competitive and cooperative interactions between *S. aureus* and *P. aeruginosa* in the CF lungs that could influence the disease outcome.

## Introduction

Cystic fibrosis (CF) is an inherited autosomal recessive genetic disorder that predominantly affects Caucasians. CF is caused due to the mutations in the cystic fibrosis transmembrane conductance regulator (*CFTR*) gene. The *CFTR* gene is present on the long arm of the chromosome 7 at the position 7q31.2. It has 27 exons and codes for the 1480 amino acid containing CFTR protein that functions as a voltage gated chloride ion channel that transports chloride ions into and out of the cells. The CFTR protein is found across the membranes of epithelial cells that line the liver, lungs, pancreas, intestines, skin and reproductive tract. CFTR in general coordinates the rate of sodium ion (Na^+^) absorption and chloride ion (Cl^−^) secretion to hydrate airway surfaces by effecting the osmotic movement of water and promotes mucus clearance (Anderson et al., 1991). To date >1000 mutations have been reported in the CFTR gene. The most common of these mutations that accounts for ~50-70% of CF cases worldwide is the in-frame deletion of phenyl alanine at position 508 (ΔF508). CFTR that lacks phenyl alanine at position 508 gets misfolded and fails to get translocated to the cell membrane and will get degraded by the quality-control mechanism of the cell (Debraekeleer and Daigneault, 1992; Bobadilla et al., 2002). In CF, the absence of a functional CFTR in the cell membranes upsets the Na^+^ and Cl^-^ ion balance that is required for maintaining the normal, hydrated thin mucus layer that can be easily cleared by cilia lining the lungs and other organs. The Na^+^ and Cl^-^ ion imbalance causes retention of water inside the cells (Anderson et al., 1991). The consequent dehydration of the extracellular space creates a thick, viscous mucus layer that cannot be cleared easily by cilia and traps bacteria resulting in chronic infections. The main characteristic feature of CF is the chronic bacterial infections of the airways and sinuses.

**Figure 1:**
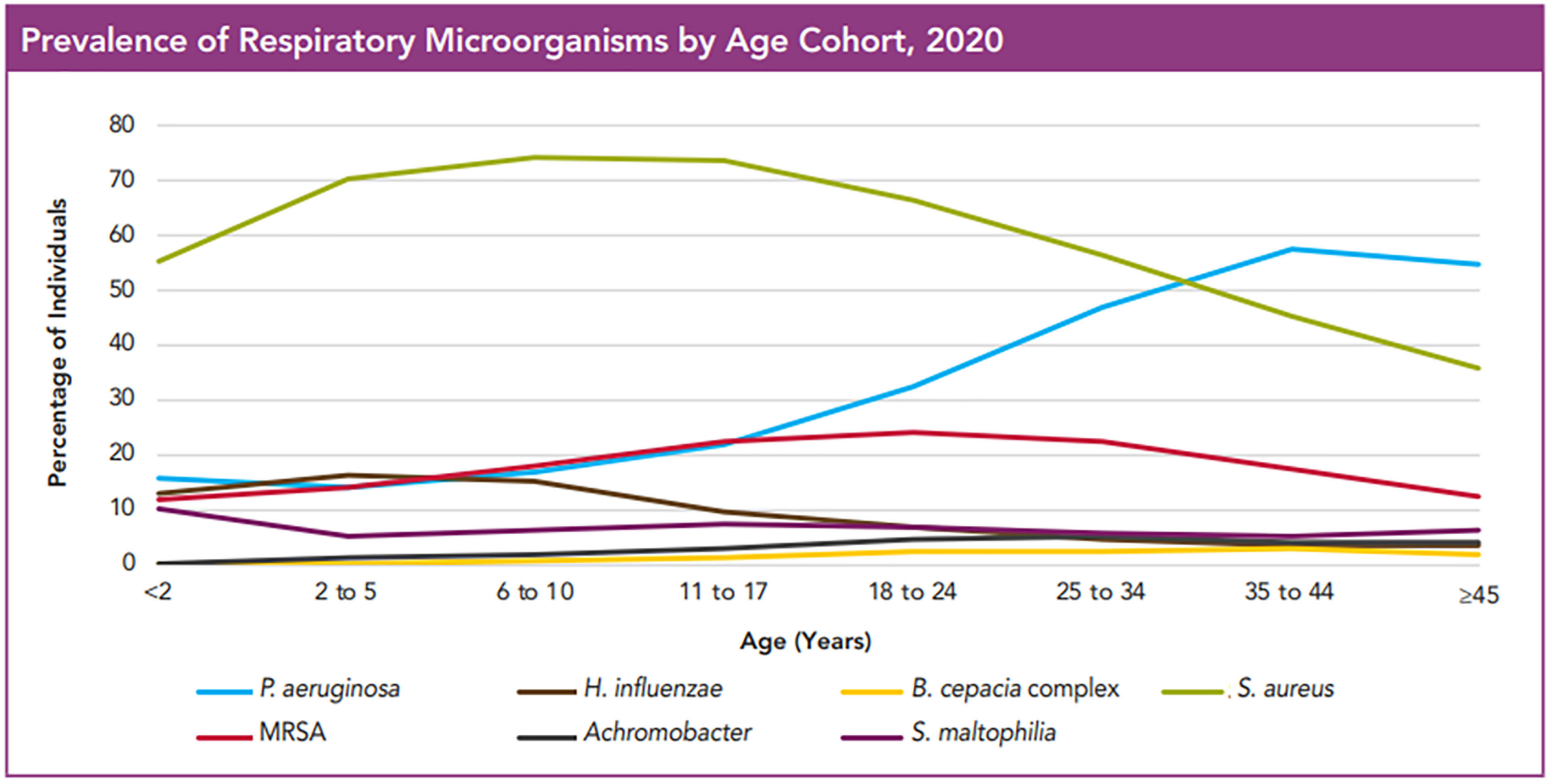
Prevalence of Respiratory Microorganisms by age cohort, 2020. The figure shows the prevalence of microorganisms in the respiratory tract of CF patients in different age groups. Even at a very young age, affected individuals have at least one microorganism, and this number increases with increasing age. *S. aureus* is by far the most widespread microorganism, which is followed with time by *P. aeruginosa*. What one can also see is that the incidence of MRSA is only about 20% of that of MSSA. *Hemophilus influencae*, *Achromobacter*, *Burkholderia cepacia* and *Stenotrophomonas maltophilia* were only rarely observed. This image is reproduced with permission from Cystic Fibrosis Foundation Patient Registry, 2020 Annual Data Report, Bethesda, Maryland^©^2021 Cystic Fibrosis Foundation.

As shown by the Cystic fibrosis foundation, patient registry, 2020 annual data report that lungs of the CF patients get colonized and infected by multiple bacteria throughout life and that the bacterial flora in the lung changes as individuals age (CysticFibrosisFoundation, 2020). More than 60% of patients harbor at least one microorganism even at very young ages, and this increases to more than 80% in older age groups. *S. aureus* is the most common microorganism across all age groups and as patients’ age it is commonly found together with *P. aeruginosa*. However, with increasing age the incidence of *S. aureus* infections declines slightly and infections with *P. aeruginosa* become equivalent or even more prominent (**Figure 1**). In addition to these two pathogens, the CF lungs were also found to be infected to a lesser extent by *Burkholderia cepacia*, *Haemophilus influenzae*, *Stenotrophomonas maltophilia* and *Achromobacter xylosoxidans* (Lyczak et al., 2002; Lipuma, 2010).

Whereas in the majority of healthy individuals, *Prevotella, Veillonella, Staphylococcus, Streptococcus, Corynebacterium, Fusobacterium*, and *Propionibacterium* were the typical genera found in the upper respiratory tract while *P. aeruginosa* rarely infects healthy human lungs (Williams et al., 2010; Clark, 2020).Exaggerated pro-inflammatory response following bacterial infections and the consequent respiratory failure are the main causes of morbidity and mortality in CF. Interactions among these bacteria greatly influence the lung function. During the early stages of infection, *P. aeruginosa* outcompetes *S. aureus* by secreting virulence products that inhibit the growth and induce the lysis of *S. aureus.* However, *S. aureus* still survives and persists in the presence of *P. aeruginosa* by employing several strategies. Several host factors have also been reported to influence the coexistence of *S. aureus* and *P. aeruginosa*. This review provides an overview of the interactions involving initial competitive, adaption and later co-operative interactions between *S. aureus* and *P. aeruginosa*, the two most common bacterial pathogens that inhabit the CF lungs. 

## Toxins of *P. aeruginosa* That Inhibit *S. aureus* Growth

*P. aeruginosa* produces a large number of virulence factors that influence the severity of CF disease. The expression of approximately 10% of the *P. aeruginosa* genome including the genes for various virulence factors and biofilm formation is dependent on its cell density-based quorum sensing (QS) systems, LasI/R, RhlI/R and *Pseudomonas* quinolone signal (PQS) systems. The LasI/R, RhlI/R cell-to-cell communication systems consists of enzymes involved in the synthesis of diffusible autoinducer *N*-acylhomoserine lactone (AHL) signal molecules and a target regulator to monitor the density of the population (Pearson et al., 2000). The PQS system involves the quinolone QS signals, 4-hydroxy-2-heptylquinoline (HHQ) and 2-heptyl-3,4-dihydroxyquinoline (PQS, pseudomonas quinolone signal (Deziel et al., 2004). With increasing cell density, these diffusible signaling molecules would accumulate and trigger coordinated responses in *P. aeruginosa* (Van Delden and Iglewski, 1998). In the LasI/R QS system, LasI directs the synthesis of *N*-(3-oxododecanoyl)-l-homoserine lactone (3-oxo-C12-HSL) for LasR activation, and in the Rhl system, RhlI, directs the production of *N-*butanoyl-L-homoserine lactone (C4-HSL) for the activation of RhlR. The QS signals of the PQS system involves derivatives of 4-hydroxy-2-alkylquinolines (HAQs) including the derivatives of HHQ and PQS that are synthesized by the enzymes PqsABCDE and PhnAB and lead to the activation of PqsR. The LasR–3-oxo-C12-HSL complex positively regulates the transcription of RhlI and RhlR. Both the Las and Rhl QS systems control the production of PQS molecules, PQS in turn regulates the expression of RhlI and RhlR (Juhas et al., 2005). Activation of these QS systems in *P. aeruginosa* leads to the production of several virulence factors such as ADP-ribosyltransferase toxins (exotoxin A and exoenzyme S), quinoline *N*-oxides, siderophores (pyochelin, pyoverdine), mucoid exopolysaccharide (alginate), endotoxin (lipopolysaccharide, LPS), phenazine pigment pyocyanin, proteases (LasA, LasB, LecA, LecB type IV protease, alkaline, and protease IV), phospholipase A2 (ExoU), and rhamnolipid etc. A number of these virulence factors that are produced by *P. aeruginosa* including the respiratory toxins (hydrogen cyanide (HCN), pyocyanin and quinoline *N*-oxides), LasA, rhamnolipids, long chain *N*-acyl homoserine lactones (AHLs) and *Cis*-2-decenoic acid inhibit the growth of *S. aureus* which are discussed below.

### Respiratory Toxins

Among the various virulence factors secreted by *P. aeruginosa* the small respiratory inhibitors such as hydrogen cyanide (HCN), pyocyanin and quinoline *N*-oxides are the most well studied; and have been found to impact greatly the interactions between the two bacteria. These factors target the electron transport chain (ETC) and suppress the growth of *S. aureus* and also modulate other competing bacterial species.

*P. aeruginosa* produces HCN by the oxidative decarboxylation of glycine by the enzyme HCN synthase that is encoded by the *hcnABC* genes (Blumer and Haas, 2000).Transcription of the *hcn* genes was found to be controlled by the quorum sensor regulators LasR and RhlR, global activator GacA, transcriptional regulator, AlgR as well as the anaerobic regulator ANR (Pessi and Haas, 2000; Pessi and Haas, 2001). The production of HCN was proven to be clinically significant in the CF lungs. Cyanide could be detected in the sputum and bronchoalveolar lavage (BAL) fluids obtained from the *P. aeruginosa*-infected CF patients, which also correlated directly with a worsening prognosis (Carroll et al., 2005; Enderby et al., 2009). *P. aeruginosa* isolates from CF patients produced higher levels of HCN compared to the laboratory strains. HCN may aid *P. aeruginosa* in eliminating other lung pathogens by blocking their respiratory chain leading to it being the dominant bacterium. The terminal cytochrome oxidase is the cellular target of HCN (Jones and Poole, 1985).The presence of a cyanide insensitive terminal oxidase encoded by *cioAB* helps *P. aeruginosa* avoid the toxic effects of cyanide produced by it. This terminal oxidase is closely related to the cytochrome *bd* quinol oxidases (Cunningham et al., 1997). HCN produced by *P. aeruginosa* blocks the transfer of electrons from the two terminal oxidases, cytochrome *o* or cytochrome *aa*3 to oxygen in the ETC of *S. aureus* and of the other staphylococcal species and inhibits their aerobic respiration (Voggu et al., 2006).

*P. aeruginosa* synthesizes copious amounts of redox active pigmented secondary metabolite, pyocyanin (PYO), whose production is regulated by PQS and Rhl quorum-sensing systems (Welsh et al., 2015). PYO acts against other commensal microbiota and is toxic to a variety of host cells. Its toxicity is attributable to the inhibition of aerobic respiration and to the generation of reactive oxygen species (ROS) such as hydrogen peroxide (H_2_O_2_) and superoxide radical $\left(O_{2}^{-}\right)$ (Hassan and Fridovich, 1980; Noto et al., 2017). PYO was found in the sputum samples obtained from the lungs of CF patients in concentrations as high as 16 µg/ml which also correlates with the progression of the disease (Wilson et al., 1988). PYO targets the *S. aureus* ETC and interferes with the transfer of electrons and diverts them to produce ROS that can induce death (Hassan and Fridovich, 1980). *P. aeruginosa* escapes the effects of PYO and its ROS by increasing the activities of the superoxide dismutase (SOD) and catalase under pyocyanin-producing conditions (Hassett et al., 1992). It is speculated that PYO inhibits the transfer of electrons either from the menaquinone pool to cytochrome *b* or from the two terminal oxidases to oxygen or it acts at both these sites (Voggu et al., 2006). Selection of *S. aureus* on PYO yielded menadione auxotrophic SCVs that were capable of growth at high concentrations of PYO (Noto et al., 2017).

*P. aeruginosa* produces a mixture of low molecular weight, hydrophobic alkyl-hydroxyquinoline N-oxides (AQNOs) that vary in the length and degree of saturation of the alkyl chains. Examples of AQNQs are 2-alkyl-4-quinolone N-oxides, 2-heptyl-4-quinolone N-oxide (HQNO) and trans-Δ(1) -2-(non-1-enyl)-4-quinolone N-oxide. The AQNOs block the oxidation of cytochrome *b* and the reduction of cytochrome *c* and in *S. aureus* they block the oxidation of cytochrome *b*1 and reduction of cytochrome *a*2 (Kogut and Lightbown, 1962; Machan et al., 1992). Several studies have demonstrated that at higher concentrations AQNOs inhibit growth and at lower concentrations they induced the selection of SCVs in *S. aureus* (Szamosvari and Böttcher, 2017).

### Staphylolysin (LasA)

*P. aeruginosa* secretes a 20-kDa metalloendopeptidase, LasA or staphylolysin that cleaves the glycyl-alanine and glycyl-glycine bonds of the pentaglycine crosslink in the peptidoglycan of *S. aureus* and induces its lysis (Lache et al., 1969; Kessler et al., 1993). *P. aeruginosa* synthesizes small, high-affinity iron-chelating compounds siderophores, pyoverdine, and pyochelin (Hoegy et al., 2014). It can also acquire iron *via* the uptake of heme molecules from the host hemoproteins. Mashburn et al. have found that *S. aureus* can be lysed by *P. aeruginosa* and that the lysed *S. aureus* cells could serve as a source of iron for *P. aeruginosa.* The gene expression patterns of the iron-regulated genes of *P. aeruginosa* were found to be similar when it is grown in the presence of *S. aureus* or in high-iron conditions indicating that *S. aureus* might serve as a source of iron for *P. aeruginosa* (Mashburn et al., 2005).

### Rhamnolipids

*P. aeruginosa* produces extracellular secondary metabolites called rhamnolipids. These are glycolipids consisting of a dimer of 3-hydroxy fatty acids that are linked *via* glycosidic bonds to one or two (L)-rhamnose molecules. The stability of the *P. aeruginosa’s* biofilm structure relies on the endogenous production of the suitable concentrations of rhamnolipids at the right time. At low concentration they increase the hydrophobicity at the cell surface and enhance aggregation of the *P. aeruginosa* cells and facilitate microcolony formation. Increased hydrophobicity also aids in augmenting the affinity of the cells initial adherence to surfaces. However, at high concentrations they decrease the surface tension and exert an anti-adhesive effect (Pamp and Tolker-Nielsen, 2007). These rhamnolipids have also been found to display anti-adhesive and biofilm dispersal activities that inhibit biofilm formation and disrupt established biofilms formed by several other bacterial and fungal species including *S. aureus* in a dose dependent manner (Wood et al., 2018).

### Long Chain N-Acyl Homoserine Lactones (AHLs)

**Figure 2:**
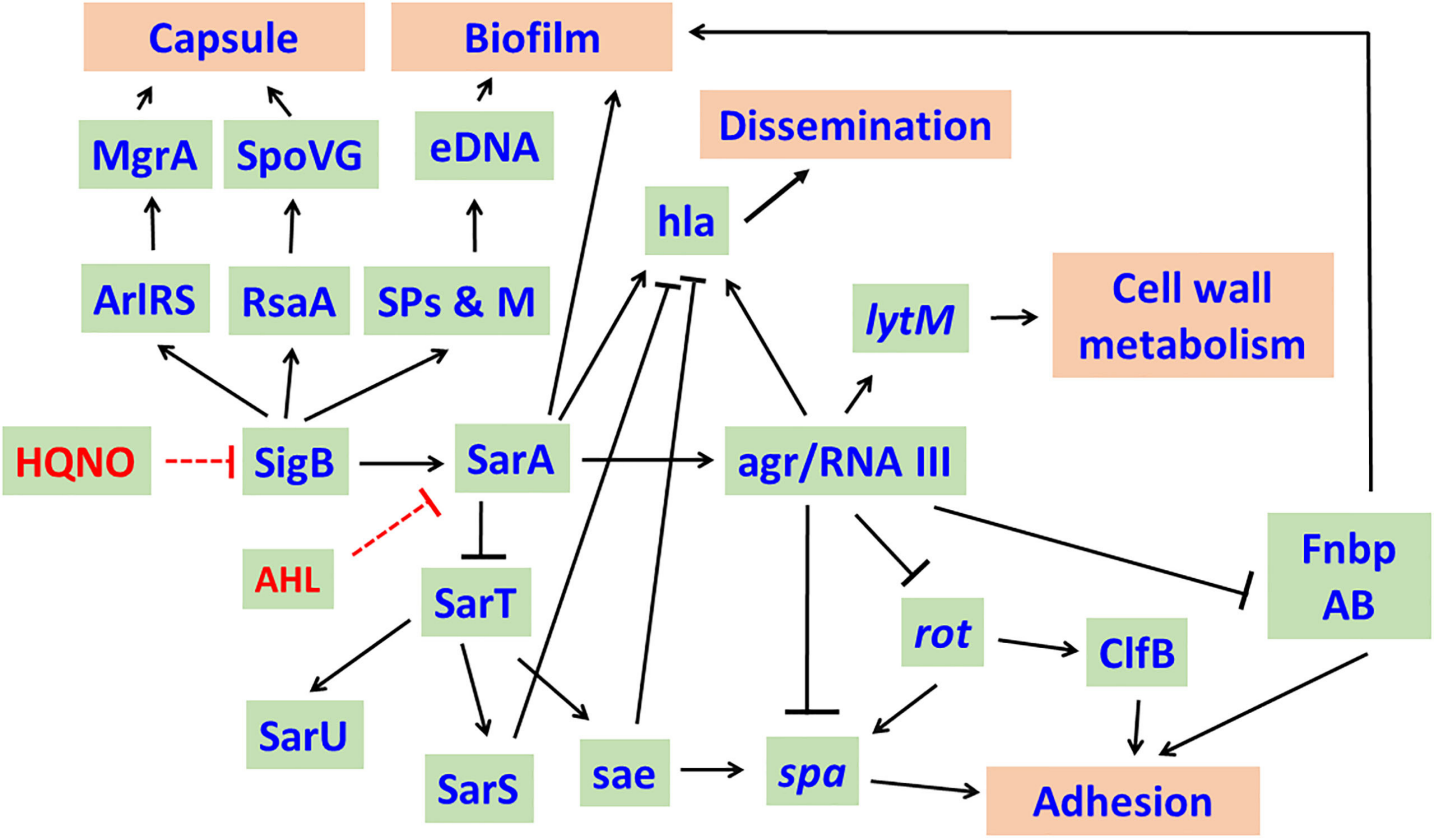
Effect of Long chain - acyl homo serine lactones (AHLs) of *P. aeruginosa* on *S. aureus*. In general, SigB and SarA regulate the expression of a number of genes and their products and enhance the virulence of *S. aureus*. AHLs of *P. aeruginosa* target SigB and SarA and cause decreased transcription of *sigB*, *sarA* and agr RNAIII resulting in decreased capsule production, biofilm formation and dissemination. Inhibition of agr RNAIII enhances the production of Protein A, ClfB, FnbpAB, which increase the adhesion of *S. aureus* to host cells. RpiRc regulates RsbU to modulate eDNA-dependent biofilm formation and *in vivo* virulence of *S. aureus* in a mouse model of catheter infection. The network was drawn based on the knowledge acquired from literature (Schmidt et al., 2003; Tamber and Cheung, 2009; Fechter et al., 2014; Andrey et al., 2015). Arrows represent activation while bars represent repression. SPs & M, Secreted proteins & metabolites; eDNA, extracellular DNA; ClfB, clumping factor B; FnbpAB, Fibronectin binding proteins A and B; *spa*, Protein A; *hla*, alpha-toxin; *sae*, *S. aureus* exoproteins.

The long chain AHL, 3-oxo-C_12_-HSL inhibits the growth of *S. aureus* in a dose dependent manner. At sub-growth inhibitory concentrations, they inhibit the expression of the staphylococcal accessory regulator (*sarA*), accessory gene regulator (*agr*) leading to the downregulation of exotoxins (hemolysins, TSST-1) production in *S. aureus*. Inhibition of SarA leads to enhanced expression of protein A (Qazi et al., 2006). Inhibition of agr in *S. aureus* by the long chain AHLs of *P. aeruginosa* results in enhanced production of surface adhesins such as fibronectin binding proteins (FnbAB) and clumping factor B (ClfB) that reduce detachment and augment robust biofilm formation and enhance host cell invasion of *S. aureus* (Schmidt et al., 2003; Qazi et al., 2006; Tamber and Cheung, 2009) (**Figure 2**).

### *Cis*-2-Decenoic Acid

*Cis*-2-decenoic acid (*cis*-DA), a small messenger molecule produced by *P. aeruginosa* that induces biofilm dispersal in a broad range of bacteria (including *P. aeruginosa* itself and *S. aureus*) and in yeast. It also has been found to enhance metabolic activity, reverse persistence, and increase the activity of antimicrobial agents. The reversal of persister cells to the antimicrobial-susceptible state by *cis*-DA has been attributed to the increased membrane permeability which allows increased uptake of antimicrobials (Marques et al., 2014). Cis-DA of *P. aeruginosa* makes *S. aureus* susceptible towards host defense system and antimicrobial agents. 

## *S. aureus* Strategies to Evade Killing by *P. aeruginosa*

It has been observed that as the patients’ age, the prevalence of *S. aureus* decreases and *P. aeruginosa* becomes the major pathogen in the adults. Most of the interaction studies have reported that during early co-infections *P. aeruginosa* outcompetes *S. aureus* by employing several strategies that range from secreting virulence products that inhibit the growth or lysis *S. aureus*. However, patients harboring both *S. aureus* and *P. aeruginosa* are detected frequently in the worldwide cohort indicating that *S. aureus* is able to survive and persist in the presence of *P. aeruginosa.* The isolation of *S. aureus* and *P. aeruginosa* from the same lobe of CF lungs suggests that they share the same ecological niche and interact *in vivo* (Hubert et al., 2013; Baldan et al., 2014). Co-existence of *S. aureus* and *P. aeruginosa* is associated with chronic infection and a faster decline in the lung function (Briaud et al., 2020). To evade *P. aeruginosa* mediated killing *S. aureus forms SCVs* and up-regulate expression of genes involved in metabolism, which we have discussed below.

### Formation of ‘Small Colony Variants’ (SCV) by *S. aureus* Evades *P. aeruginosa* Mediated Killing

**Figure 3:**
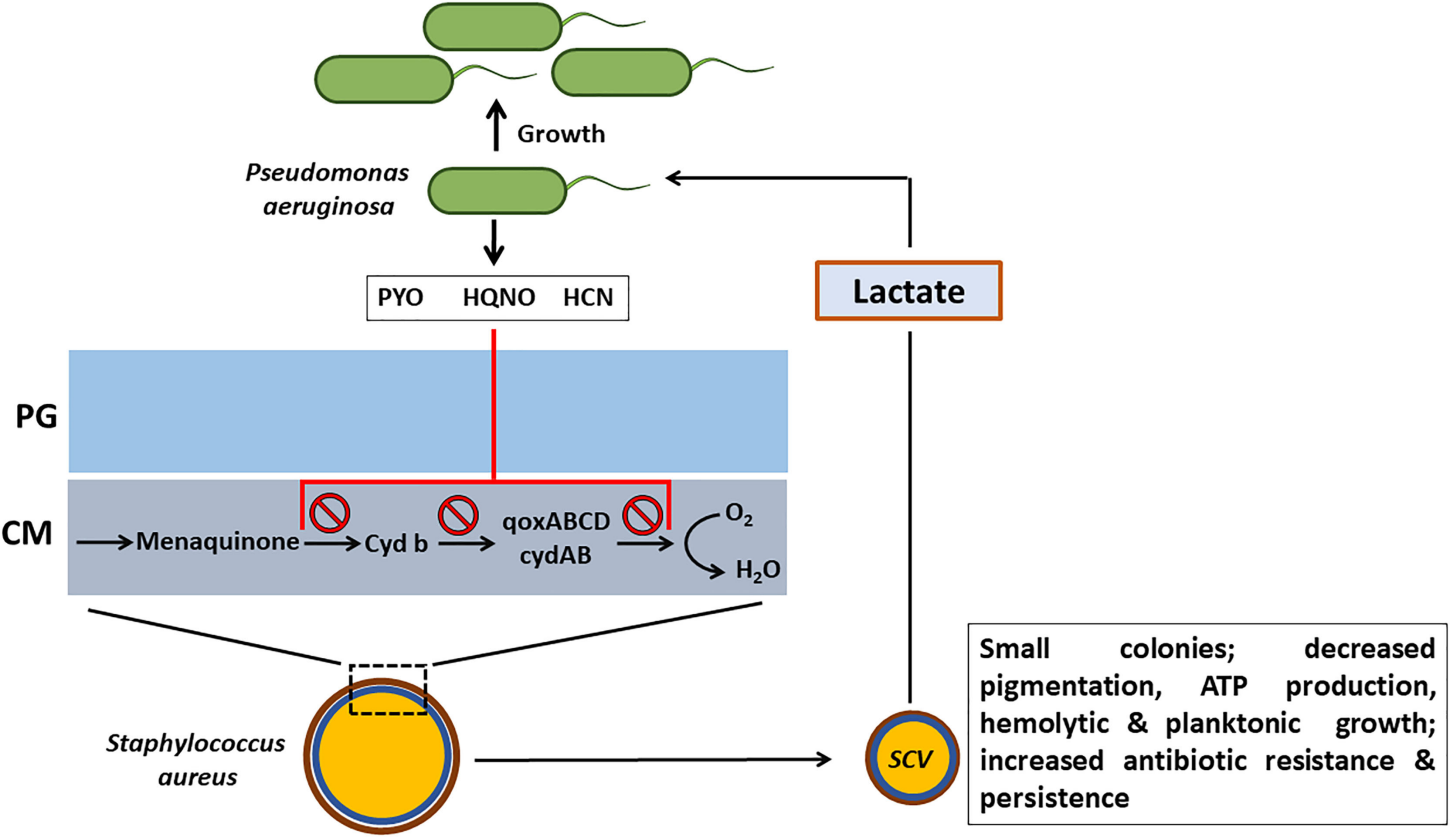
Effect of respiratory inhibitors of *P. aeruginosa* on *S. aureus. P. aeruginosa* secretes small respiratory inhibitors such as pyocyanin (PYO), hydrogen cyanide (HCN) and quinoline *N*-oxides (HQNO) that target the electron transport pathway (ETC) and block the transport of electrons. *S. aureus* shifts to fermentative metabolism and adapts the small colony variant (SCV) phenotype to survive. In addition to resisting the respiratory inhibitors of *P. aeruginosa*, SCV phenotype imparts several advantages to *S. aureus* such as increased antibiotic resistance and persistence. The lactate that is produced by *S. aureus* SCVs will be consumed by *P. aeruginosa* as a preferential carbon source. PG, peptidoglycan; CM, cell memebrane.

Despite the presence of the respiratory inhibitors (HCN, PYO and quinoline *N*-oxides) produced by *P. aeruginosa* that block the transfer of electrons in the ETC and suppress its growth, *S. aureus* survives the respiratory attack and sustains infection by adapting to respiration-defective small colony variant (SCV) phenotype (Hoffman et al., 2006; Biswas et al., 2009). These SCVs cause persistent and recurrent infections and are also resistant to antibiotics such as aminoglycosides, antifolate antibiotics, and to host antimicrobial peptides such as HBD2 and HBD3, LL-37 and lactoferricin B (**Figure 3**). SCVs also exhibit enhanced host cell invasion and intracellular survival, robust biofilm formation, and increased capsule production through a combination of enhanced SigB activity and decreased Agr activity that results in overexpression of surface proteins and insufficient cytolysin production (Moisan et al., 2006; Mitchell et al., 2010). SCVs of *S. aureus* grow as tiny, non-hemolytic, non-pigmented colonies and are usually auxotrophic to thymidine, menadione or hemin, or can be of transient SCV phenotype that can revert back to the wild-type phenotype in the absence of the selection pressure (Besier et al., 2007). Thymidine auxotrophic *S. aureus* SCVs were found to be induced by long-term Trimethoprim-Sulfamethoxazole (SXT) treatment and have increased fitness during SXT Challenge. These SCVs fail to synthesize DNA due to mutations in the thymidylate synthase gene (Kriegeskorte et al., 2015).

Genome sequencing of the menadione auxotrophic *S. aureus* SCV that grew in the presence of high concentrations of pyocyanin led to the identification of mutations in four genes, *qsrR, saeS*, *menD*, and NWMN_0006 (Noto et al., 2017). The *qsrR* gene encodes for a quinone-sensing and response repressor of quinone detoxification genes whose inactivation results in the over-expression of quinone/ROS detoxification enzymes and significant pyocyanin resistance (Ji et al., 2013). The *saeS* gene encodes for the sensor histidine kinase SaeS which is part of the SaeRS two-component system (TCS) that comprises of SaeS along with the response regulator SaeR. The TCS SaeRS plays a vital role in controlling the production of more than 20 virulence factors including surface proteins, proteases, hemolysins, leukocidins and superantigens (Schäfer et al., 2009). The *menD* gene encodes for the enzyme 2-succinyl-5-enolpyruvyl-6-hydroxy-3-cyclohexene-1-carboxylate synthase [NWMN_0913]), which is necessary for menaquinone biosynthesis.

In addition to the blocking of the electron transport chain, PYO also exerts its toxicity through the generation of ROS. *S. aureus* in general uses superoxide dismutases (SodA/M), catalase (KatA), and the pigment staphyloxanthin to combat ROS such as $O_{2}^{-}$ and H_2_O_2_. However, the production of staphyloxanthin by the *S. aureus* SCVs is significantly reduced and also heme auxotrophs are deficient in catalase. Interestingly, exposure of wild-type *S. aureus* to H_2_O_2_ consistently led to the generation of menadione auxotrophs. The appearance of SCVs from the H_2_O_2_ exposure involved the SOS response that includes DNA double-strand break repair pathway proteins RexAB, polymerase V, and recombinase A (Painter et al., 2015). Clinical menadione-auxotrophic SCV isolates demonstrated increased H_2_O_2_ resistance with elevated catalase activity, relative to a wild-type revertant. Also, the redox-sensing regulator, Rex aids the respiration deficient *S. aureus* SCVs in avoiding the redox stress by shifting to fermentative metabolism (Pagels et al., 2010; Filkins et al., 2015). The fermentative metabolism also imparts resistance to SCVs against ROS mediated killing by decreasing the need for metabolic enzymes and cytochromes that require iron as a cofactor. Metabolic analysis of the *S. aureus* SCVs has revealed significant increase in the production of lactate, the primary source of energy generation in the fermentative metabolism. *P. aeruginosa* was found to preferentially consume the *S. aureus*-produced lactate as a carbon source over the medium-supplied glucose (**Figure 3**).

### L-Form-Like *S. aureus* Cells Evade LasA Mediated Lysis by *P. aeruginosa*

**Figure 4:**
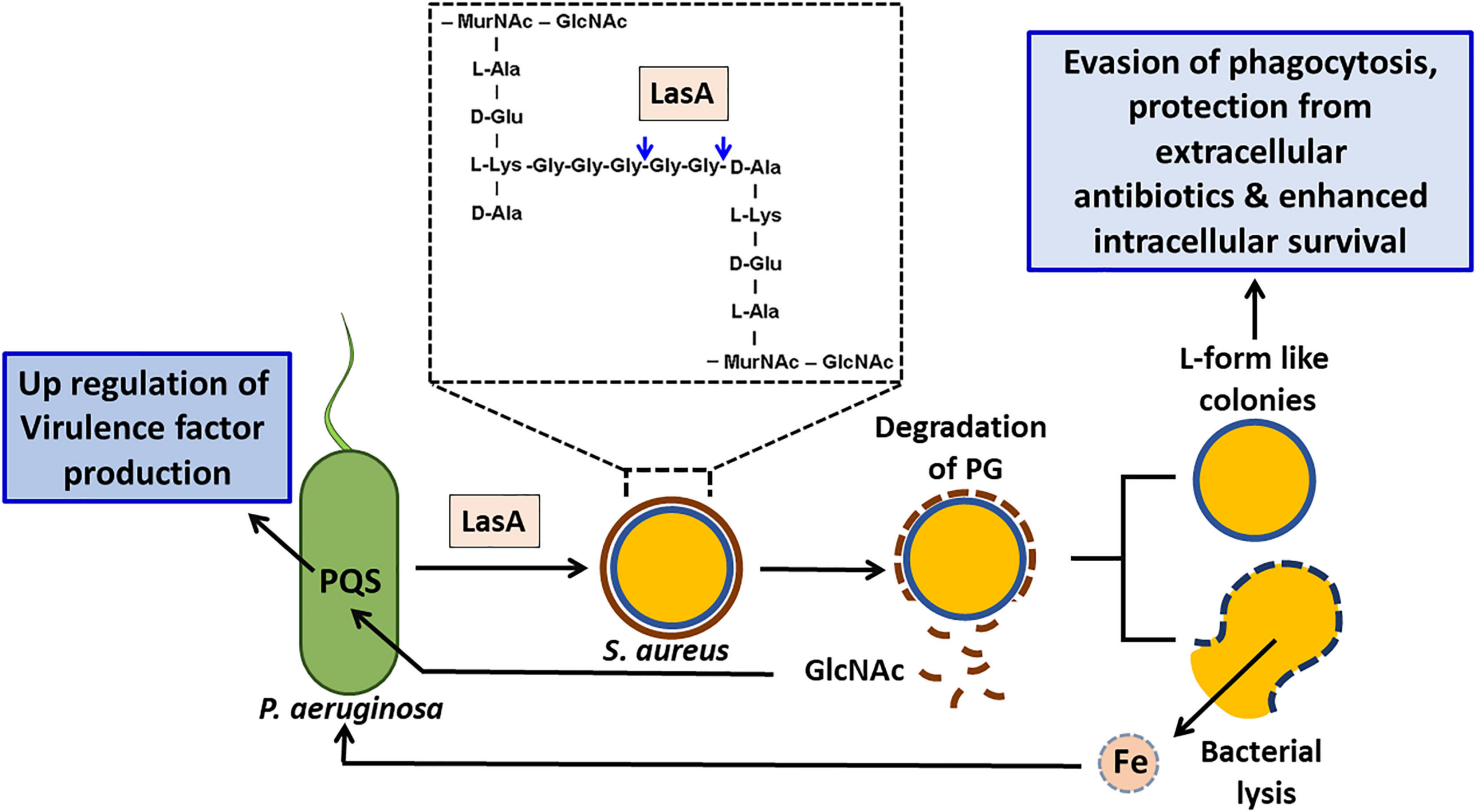
Effect of staphylolysin (LasA) of *P. aeruginosa* on *S. aureus. P. aeruginosa* secreted staphylolysin (LasA) targets the glycyl-glycine and glycyl-alanine bonds of the pentaglycine cross-linkage in the peptidoglycan of *S. aureus* and induces its lysis. Lysed *S. aureus* cells could serve as a source of iron for *P. aeruginosa* thus promotes its growth. The *N*-acetyl glucosamine (GlcNAc) that is shed will be sensed by *P. aeruginosa* and enhances its PQS quorum sensing system and induces the production of extracellular virulence factors such as PYO, rhamnolipids and LasA etc that will aid in eliminating its competitors. *S. aureus* adapts the L-form like colonies to survive. In addition to surviving the effects of LasA, L-form like colonies confer several advantages to *S. aureus* such as evasion of phagocytosis, enhanced intracellular survival and protection from the effect of extracellular antibiotics.

*S. aureus* survives the activity of LasA produced by *P. aeruginosa* by forming the cell wall deficient L-form-like colonies (Falcon et al., 1989). *S. aureus* as L-forms were found to internalize, replicate and persist in the lungs of the infected rats by preventing the formation of phagolysomes and digestive vacuoles (Schmitt-Slomska et al., 1986; Michailova et al., 2007). Intracellular persistence as L-forms would provide *S. aureus* with an opportunity to not only evade professional phagocytes but also protect it from extracellular antibiotics and would promote recrudescent infection (**Figure 4**). 

### *S. aureus* Alters Expression of Metabolic Genes to Encounter Killing by *P. aeruginosa*

In competitive interactions the major metabolic pathways such as Krebs cycle (downregulation of Acetyl-coA synthetase resulting in reduced ATP production), translation (increased levels of tRNAs and ribosomal RNAs are associated with reduced translation efficiency) and oxidative stress of *S. aureus* were found to be affected. Upregulation of several dehydrogenase enzymes, such as lactate dehydrogenase (*ldhA*), glutamate dehydrogenase (*gluD*), alanine dehydrogenase (*ald1*), 2-oxoglutarate dehydrogenase (*odhA*), 1-pyrroline-5-carboxylate dehydrogenase (*rocA*) and aldehyde-alcohol dehydrogenase (*adhE*) as well as the L-lactate permease (*lctP*) suggests a switch to lactic acid fermentation from aerobic respiration. *P. aeruginosa* consumes the lactate generated by *S. aureus* as a carbon source. The *adhE* and *gluD* are also associated with oxidative stress responses (Briaud et al., 2019). Additionally, the genes *pgi* (glucose-6-phosphate isomerase), *fbp* (fructose-bisphosphate aldolase) and *fda* (fructose-1,6-bisphosphatase) that were involved in glycolysis and pentose phosphate pathways were down-regulated indicating a competition for nutrients and that *S. aureus* uses sources other than glucose to produce energy and nucleotides.

The presence of *P. aeruginosa* also effects the expression of the genes involved in the nucleotide metabolism in *S. aureus*. The two *nrd* operons (*nrdE*, *nrdI*, *nrdF* and *nrdG*, *nrdD*) that regulate the production of deoxyribonucleotide di- or triphosphates were down-regulated while the *deoD* gene that encodes for purine nucleoside phosphorylase which plays a major role in alternative metabolic pathway for nucleotides was upregulated (Briaud et al., 2019). As the *nrd* operons play a vital role in DNA synthesis and its repair control, limiting the synthesis of deoxyribonucleotide phosphates will affect the cell concentration.

In response to the co-culture induced nitrogen source limitation particularly purines, urea, and amino acids (mostly glutamine), *P. aeruginosa* up-regulated its master nitrogen regulator gene, *ntrC* and other genes involved in nitrogen assimilation such as *gdhA* (glutamate dehydrogenase), *glnA* (glutamine synthase) and *ureE* (urease accessory factor). Also the expression of the glutamine amido/aminotransferase encoding operon PA14_06890-PA14_06930 operon was enhanced in the presence of *S. aureus*. While in response to nitrogen starvation *S. aureus* reduced the expression of its glutamate synthase encoding genes, *gltB* and *gltD* that convert glutamine to glutamate. Moreover, increased expression of glutamine binding protein (NWMN_1750) in *S. aureus* and ammonium transporter (PA14_24780) in *P. aeruginosa* indicate that both bacteria try to acquire nitrogen from extracellular sources. These modifications suggest that co-existence induces competition for nutrient resources and triggers global responses that are mostly dominated by metabolic adaptations (Tognon et al., 2019). In response to the stress caused due to nutrient limitation or cell damage both these pathogens were found to trigger lysogenic mechanisms as was evidenced by the induction of the prophages in *S. aureus* and R- and F- pyocin synthesis genes in *P. aeruginosa* (Tognon et al., 2019). R-type, S-type, and F-type pyocins are bacteriocins that are secreted by *P. aeruginosa* into the environment to reduce competition from other bacterial strains. They differ in their morphology and mode of killing (Nakayama et al., 2000). The cell lysis resulting from the induction of pyocin and prophages in a small sub-population of the culture could aid in sensing the presence of a resource competitor. *P. aeruginosa* also has the ability to sense the peptidoglycan component *N*-acetylglucosamine (GlcNAc) that is shed by the Gram-positive commensal flora during cell lysis or cell wall turnover. Community surveillance by sensing GlcNAc aids *P. aeruginosa* in eliminating its competitors by enhancing the PQS QS induced production of virulence factors such as PYO, rhamnolipids and LasA etc (Korgaonkar et al., 2013).

### In Coexistence With *S. aureus*, *P. aeruginosa* Loses Virulence

During co-existence with *P. aeruginosa*, *S. aureus* employs several strategies to survive the attack of *P. aeruginosa* such as formation of SCVs and altering the expression patterns of several of its genes. Recently it was found that *S. aureus* also influences the overall virulence of *P. aeruginosa*. The strains of *P. aeruginosa* coexisting with *S. aureus* were found to be less aggressive compared to the strains isolated from the mono-infected patients which were more competitive towards *S. aureus* and exhibited antagonistic interactions. It was observed that the early and late clonal variants of *P. aeruginosa* adopt different phenotypes that influence the growth of *S. aureus* differently. While the early-infecting *P. aeruginosa* strains had a negative effect on *S. aureus* growth and had competitive interactions, *P. aeruginosa* strains in the later stages of chronic infection exhibited significantly attenuated virulence towards *S. aureus*. During chronic infections in the process of adapting to the CF lungs, *P. aeruginosa* undergoes microevolution that results in the acquisition of mucoidy, enhanced antibiotic resistance, loss of motility, and loss-of function mutations in virulence genes (Baldan et al., 2014). *P. aeruginosa* isolates with the mucoid phenotype were found to be the least competitive among the co-isolates. Acquisition of the mucoid phenotype results from the loss-of-function mutation within the *mucA* gene that encodes for an anti-sigma factor that usually prevents the synthesis of alginate, a polymer of *O*-acetylated D-mannuronic acid and L-guluronic acids. The loss-of-function mutation within the *mucA* gene was found to downregulate the transcriptionof the gene *pvdA* that leads to reduced production of a subset of virulence factors such as rhamnolipids, pyoverdine, and 2-heptyl-4-hydroxyquinolone-*N*-oxide (HQNO) that act against *S. aureus.* Exogenous alginate thereby prevents the killing of *S. aureus* and promotes the coexistence of *P. aeruginosa* with *S. aureus* within the CF lung (Limoli et al., 2017; Price et al., 2020). Interestingly, exogenous alginate was found to protect both planktonic cells and biofilms of *S. aureus* from antibiotics suggesting a shift from competitive to cooperative interactions between the two pathogens (Orazi and O’Toole, 2017).

## Co-Operative Interactions Between *S. aureus* and *P. aeruginosa*

**Figure 5:**
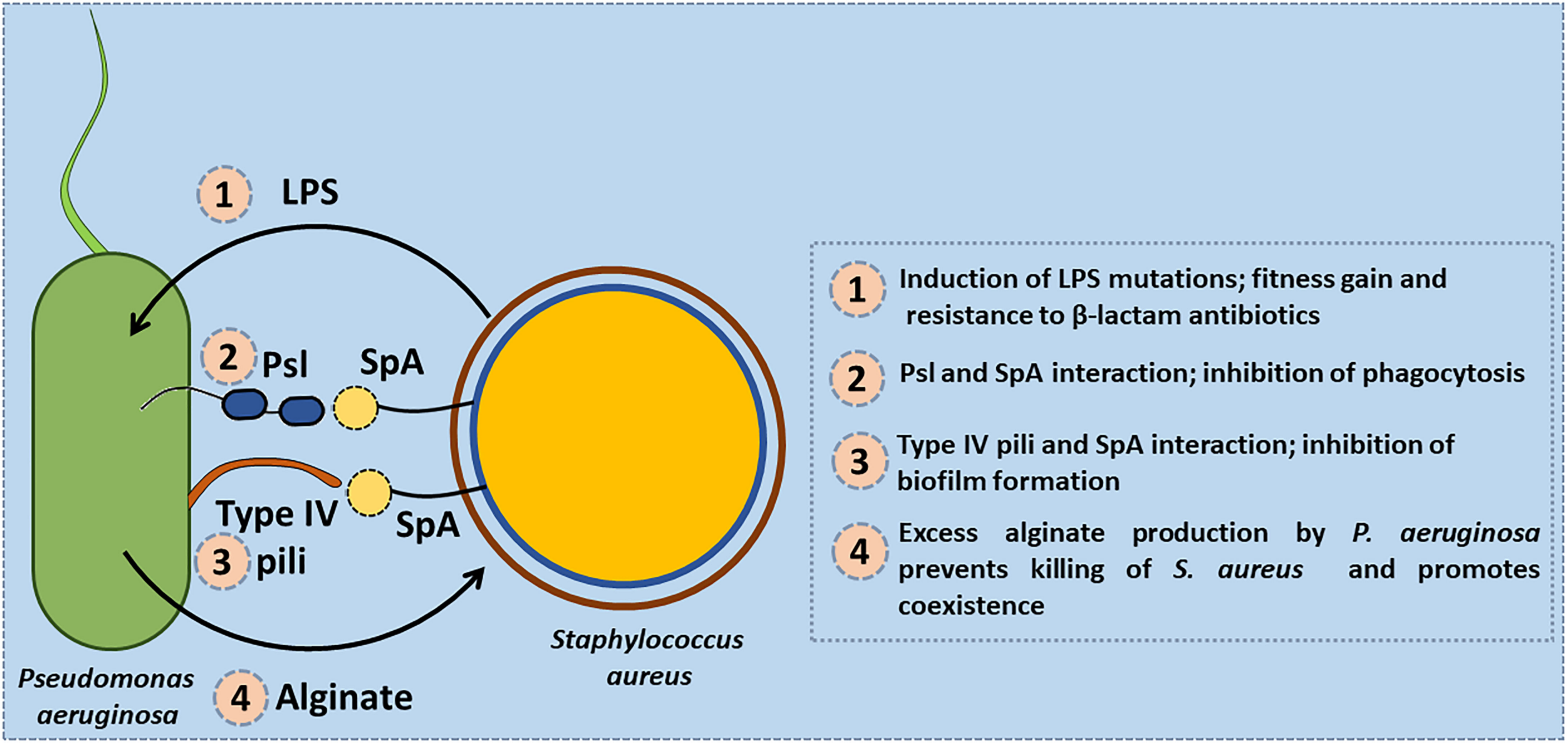
Co-operative interactions between S. aureus and P. aeruginosa. (1) *S. aureus* induces mutations in the genes encoding the lipopolysaccharide (LPS) biosynthesis in *P. aeruginosa* that confers fitness gain and enhanced resistance to β-lactam antibiotics. (2) Protein A (SpA) produced by *S. aureus* interacts with the Psl (*Pseudomonas* polysaccharide locus) polysaccharide of *P. aeruginosa* and protects it from being phagocytosed by neutrophils. (3) SpA interacts with type IV pili of *P. aeruginosa* and inhibits biofilm formation by *P. aeruginosa* strains. (4) Exogenous alginate secreted by *P. aeruginosa* protects *S. aureus* by reducing the production of virulence factors such as siderophores, and rhamnolipids.

In addition to these competitive interactions several co-operative interactions between *S. aureus* and *P. aeruginosa* have also been reported. When *S. aureus* was co-cultured with *P. aeruginosa* it was found to induce mutations in the genes encoding the lipopolysaccharide (LPS) biosynthesis which confers fitness gain and is also associated with enhanced resistance towards β-lactam antibiotics (Tognon et al., 2017). Exoproducts of *S. aureus* were also found to restore and enhance swimming and swarming motility of *P. aeruginosa* in an isolate dependent manner (Pallett et al., 2019). Protein A produced by *S. aureus* exists in both secreted and membrane-bound forms. The secreted Protein A interacts with two specific structures on the cell surface of *P. aeruginosa*, the Psl (*Pseudomonas* polysaccharide locus) polysaccharide and the PilA protein component of type IV pili. In the absence of Psl, Protein A binds to type IV pili and inhibits biofilm formation by *P. aeruginosa* strains. Protein A binds the Fcγ domain of the immunoglobulin and prevents Fc receptor mediated opsonophagocytosis and killing of *S. aureus* (**Figure 5**). It is found to impart similar protection to *P. aeruginosa* from IgG-mediated neutrophil opsonophagocytosis *in vitro* (Armbruster et al., 2016). HQNO and PQS molecules secreted by *P. aeruginosa* were found to enhance biofilm formation by *S. aureus* (Fugere et al., 2014).

## Influence of Host Factors on Coexistence of *S. aureus* and *P. aeruginosa*

*S. aureus* is the main colonizing bacteria of the CF lungs during infancy and early childhood, its incidence declines thereafter and infections by *P. aeruginosa* become more prominent with increasing age. Reduced mucociliary clearance, over-sulfation of the glycocalyx, reduced sialylation of apical proteins and increased concentration of asialoganglioside 1 (aGM1) in apical membranes of CF epithelia likely aid in the adherence and subsequent colonization of *S. aureus* and *P. aeruginosa* and contribute to the pathogenesis of these bacterial infections in the CF lung (Cheng et al., 1989; Michailova et al., 2007). Altered ionic composition, volume and pH of the airway surface liquid (ASL) due to defective CFTR function play critical roles in the pathophysiology of CF (Verkman et al., 2003). Abnormally low pH of the ASL in the CF bronchial epithelial cells was found to play a significant role in the initial bacterial colonization of the *S. aureus* within the first hours of life. Defective cAMP-dependent bicarbonate secretion that involves CFTR and SLC26A4 (pendrin) and persistent H^+^ secretion by ATP12A have been found to play a role in the abnormal acidification of the ASL. The major antimicrobial peptides (AMPs) in the lung tissues and secretions are cathelicidin LL-37, neutrophil α-defensins/Human Neutrophil Peptides, HBDs, Secreted group IIA phospholipase A2 (sPLA2-IIA) (Hiemstra et al., 2016). In the healthy lungs, these AMPs play a critical role in protection against infection by pathogens. In CF lungs, the pH of the ASL compromises the activity of the AMPs such as LL-37 and HBD1 which is vital for *S. aureus* clearance from the airways (Simonin et al., 2019). The abnormally high NaCl concentration in the CF ASL also interferes with the activity of AMPs such as HBD-1 (Goldman et al., 1997). The predominance of *S. aureus* in lung expectorate early in life of the CF patients could be related to the CF-specific defective immune response that is dependent on the pH and ionic composition of the CF ASL. *P. aeruginosa* has been reported to manipulate the host immune response by inducing strong expression of the cationic enzyme, sPLA2-IIA *via* a Krüppel-like factor 2 (KLF2) transcription factor dependent pathway. Of the four type-III secretion system (T3SS) effector molecules (ExoS, ExoT, ExoU and ExoY) produced by *P. aeruginosa*, ExoS, has been found to be involved in the induction of the expression of sPLA2-IIA by the host cells (Belmadi et al., 2018). The levels of the sPLA2-IIA produced are adequate to kill *S. aureus* with little effect on *P. aeruginosa* (Pernet et al., 2014). This could be a possible contributing factor for the shift in the infections from *S. aureus* in the early stages of CF to *P. aeruginosa* as the major pathogen in the later stages.

Anoxia arising due to static mucus, reduced pulmonary function and consumption of oxygen by host neutrophils facilitates co-existence of *S. aureus* and *P. aeruginosa* in the CF lung. Under anoxia most *P. aeruginosa* CF isolates were unable to antagonize the growth of *S. aureus* (Pallett et al., 2019). Physiological concentration of serum albumin was shown to promote coexistence of *S. aureus* and *P. aeruginosa* by inhibiting quorum sensing in *P. aeruginosa* by sequestering its AHLs and repressing the production of virulence factors that can kill *S. aureus* (Smith et al., 2017). Additionally, the host innate immune protein calprotectin was shown to promote co-existence of *S. aureus* and *P. aeruginosa* by sequestering zinc- and manganese metal ions and repressing the production of anti-staphylococcal compounds by *P. aeruginosa* (Wakeman et al., 2016). Volatile molecules present in the BAL fluid might aid in ruling in *P. aeruginosa* and ruling out *S. aureus* infections in CF patients (Nasir et al., 2018).

## The Interactions of *S. aureus* and *P. aeruginosa* Leads to Increased Antimicrobial Resistance

Interspecies interactions within the microbial community in the CF lungs influence the efficacy of different antibiotics. Both *P. aeruginosa* and *S. aureus* have the ability to form biofilms and exhibit intrinsic and acquired resistance to antibiotics which makes treating their infections difficult. In the CF lungs both these pathogens adapt a biofilm-like growth as the hypoxia and the thick, dehydrated mucus provide optimal conditions for biofilm formation. Encased within their extracellular polysaccharide matrices these pathogens exhibit 100-1000-fold more resistance to antibiotics and host immune responses compared to their planktonic counterparts because of poor penetration and slow growth (SCV formation). *P. aeruginosa* secreted HQNO, PYO and HCN block the ETC in the *S. aureus* which in turn resorts to SCV formation by shifting to fermentative metabolism resulting in decreased ATP production (Hoffman et al., 2006; Voggu et al., 2006; Biswas et al., 2009; Götz and Mayer, 2013). Reduced ATP negatively impacts the active transport which is essential for the a minoglycoside antibiotics to gain entry into the cells. These SCVs display enhanced tolerance to several antimicrobial agents (Lechner et al., 2012). Similarly, the presence of *S. aureus* was also found to promote the selection of SCVs in *P. aeruginosa* that exhibit increased resistance to several antibiotics (Michelsen et al., 2014). These interactions during coexistence were found to be mediated by the *agr* quorum sensing system, the global regulator SarA and the ClpP proteolytic complexes which regulate the expression of major virulence factors in *S. aureus*.

The presence of *P. aeruginosa* was found to induce the over-expression of *nor* genes (*tet38*, *norA*, and *norC*) in *S. aureus.* The efflux pump proteins encoded by these *nor* genes are responsible for the increased antibiotic resistance against tetracycline and fluoroquinolone (ciprofloxacin). They also promote internalization of *S. aureus* by the host epithelial cells (Briaud et al., 2019). LasA endopeptidase and rhamnolipids produced by *P. aeruginosa* enhanced the killing of *S. aureus* by vancomycin and tobramycin, by enhancing lysis and facilitating proton-motive force-independent uptake, respectively (Radlinski et al., 2017). In mixed species biofilms, *P. aeruginosa* decreased the susceptibility of *S. aureus* to a broad range of antimicrobials such as tetracycline, vancomycin, ampicillin and ceftriaxone while increasing the sensitivity towards quinolones through the secretion of metabolites such as HQNO and siderophores - pyoverdine and pyochelin (Orazi and O’Toole, 2017). *S. aureus* in mixed cultures was also found to increase the sensitivity of *P. aeruginosa* to ciprofloxacin and aminoglycosides antibiotics by tenfold compared to the monocultures (Trizna et al., 2020). The protein A of *S. aureus* was shown to increase the resistance of *P. aeruginosa* to inhaled tobramycin. In the presence of *S. aureus, P. aeruginosa* synthesizes truncated LPS that lack O-specific antigen (OSA) which imparts more resistance to β-lactam antibiotics but not against ciprofloxacin or polymyxin (Tognon et al., 2017). These studies indicate that in mixed biofilms *P. aeruginosa* acquires resistance against antibiotics that target mostly the cell wall and protein biosynthesis but the other resistance mechanisms might remain largely unaffected. 

## Interaction of P. *aeruginosa* With Other Staphylococcal Species

Earlier studies have shown that *S. aureus* has three different terminal cytochrome oxidases, the heme/copper oxidases [cytochrome *aa*3 (QoxABCD) and cytochrome *o*] and the heme-only cytochrome *bd* quinol oxidase (CydAB) (Faller et al., 1980; Voggu et al., 2006; Götz and Mayer, 2013; Hammer et al., 2016).The respiratory inhibitors secreted by *P. aeruginosa* exert their toxic effects not only on *S. aureus* but also on the other pathogenic staphylococcal species including *S. epidermidis*, *S. haemolyticus*, *S. hyicus*, *S. lugdunensis, S. muscae* and *S. saprophyticus.* Interestingly, the non-pathogenic staphylococcal species such as *S. carnosus*, *S. gallinarum, S. lentus, S. piscifermentans* and *S. simulans* are resistant to these toxins. The resistance of the non-pathogenic staphyloccal species lies in the presence of a functional alternative terminal oxidase, the pyocyanin and cyanide resistant cytochrome *bd* quinol oxidase, (CydAB). It oxidises ubiquinol and reduces oxygen as part of the ETC and is composed of two subunits, CydA and CydB. While both pathogenic and non-pathogenic staphylococci harbour CydAB terminal oxidase, the sequence of the CydA was found to be more conserved than CydB among staphylococci (Voggu et al., 2006). The structural alterations in the CydB have been reported to govern the impact of the respiratory inhibitors to block respiration and thereby influence the viability of the pathogenic staphylococci (Voggu et al., 2006). The microevolution of the CydB could possibly be due to the fact that the non-pathogenic staphylococci might have been under pressure for selection of a greater resistance to the respiratory toxins as they frequently inhabit the same ecological niche as Pseudomonas species than the pathogenic staphylococci (Voggu et al., 2006).

Disruption of the QS mediated communication of the *P. aeruginosa* by the quorum quenching enzymes and quorum sensing inhibitors (QSI) could lead to decreased virulence factor production. *Staphylococcus intermedius*, *S. delphini*, *S. pseudintermedius*, *S. lutrae* and *S. schleiferi* are the coagulase-positive bacterial species that belong to the *Staphylococcus* intermedius group (Fitzgerald, 2009; Ben Zakour et al., 2012). They are primarily zoonotic pathogens and rarely cause severe zoonotic infections of humans. These staphylococcal species were also found to secrete the so-called trace amines (TAs) like phenethylamine, tyramine, or tryptamine which boost internalization by host cells and interact with adrenergic receptors (Luqman et al., 2018; Luqman et al., 2019). Urea derivatives of trace amines, like N-[2-(1H-indol-3-yl)ethyl]-urea and N-(2-phenethyl)-urea, which were named yayurea A and B, suppress the virulent factor production in not only *P. aeruginosa* but also in other Gram-negative bacteria such as *Vibrio harveyi*, *Serratia marcescens*, and *Chromobacterium subtsugae* (Chu et al., 2013). While the yayurea A and B do not inhibit growth their QS inhibiting effects suppress the QS dependent virulent factor production including the production of PYO and other respiratory toxins in *P. aeruginosa*. When yayurea A and B were added to the medium *S. aureus* was protected from the growth inhibition by *P. aeruginosa* without the former having to undergo physiological transformation into the SCV (Chu et al., 2013).

Many Gram-negative bacteria employ *N*-acylhomoserine lactone (AHL)-mediated quorum sensing to control virulence. But surprisingly *S. aureus* is also responsive to AHLs of Gram-negative bacteria (Qazi et al., 2006). Recently it has been shown that coagulase-negative staphylococci (CNS) such as *S. carnosus*, *S. haemolyticus*, *S. saprophyticus* and *S. sciuri* secrete a *N*-Acylhomoserine lactonase (AHL-lactonase, *ahlS*) that is able to degrade *N*-Acylhomoserine lactones in *P. aeruginosa* thus causing a decrease in PYO and elastase production (Morohoshi et al., 2020). This leads us back to our earlier assumption that *S. aureus* is less protected from *Pseudomonas*, than many CNS species which have PYO and cyanide resistant terminal cytochrome oxidase (Voggu et al., 2006). To survive the respiratory toxins *S. aureus* shifts, or mutates, to SCVs (Biswas et al., 2009). Despite this disadvantage that *S. aureus* has over *P. aeruginosa*, it is amazing how persistently *S. aureus* can colonize lungs of CF patients over a long period of time, alone or together with *P. aeruginosa*. In contrast to many free-living CNS, *S. aureus* may not have learned to defend itself adequately against *Pseudomonas*. Presumably, *S. aureus* did not need to do so, since the CF lung is one of the very few habitats in which it encounters *P. aeruginosa*. 

## Discussion

Airways of patients with CF are chronically colonized with complex, polymicrobial infections. *S. aureus* is usually detected at early stages and *P. aeruginosa* becomes predominant at later stages. A number of studies reported antagonistic interactions between these two pathogens. However, clinical data have also documented the co-existence of *P. aeruginosa* and *S. aureus* in CF lungs. In this review we aimed to detail the mechanisms of *P. aeruginosa* and *S. aureus* interactions in the CF environment. *P. aeruginosa* produces a number to toxins that are active against *S. aureus*. However, in the *in vivo* CF lung, *P. aeruginosa* may become less aggressive toward *S. aureus*. Overall, three major mechanisms of interaction between *P. aeruginosa* and *S. aureus* were observed: (i) the initial interactions in which *P. aeruginosa* express a number of virulence factors such as PYO, LasA or rhamnolipids to outcompete *S. aureus*, (ii) adaptation of *S. aureus* to SCV and L-forms through phenotypic and genetic modulations to ensure their survival and co-exist in the CF lungs with *P. aeruginosa*, (iii) adaptation of *P. aeruginosa* to the CF environment alters its QS networks and reduces its virulence factor production and promotes co-existence with *S. aureus*. The modulation of the CF lung environment by *P. aeruginosa* promotes *S. aureus* colonization. Activation of NF-κB by *P. aeruginosa* LPS and increased production of TNF-α induced by *P. aeruginosa* was found to increase *S. aureus* invasion in murine lung infection models (Millette et al., 2019). *P. aeruginosa* infections could also increase the expression of host cell components, ITGA-5 and ICAM-1 which further facilitate *S. aureus* interactions with host epithelial and endothelial cells in the lung environment. Furthermore, *S. aureus* secreted proteins protect *P. aeruginosa* from opsonophagocytic killing by host. These overall interactions lead to excessive lung damage and increased antibiotic resistance in both these pathogens. This review summarized the information available so far regarding *P. aeruginosa* with *S. aureus* interactions in CF lung, however further studied need to be performed for complete understanding of the complex micro-environment present within the CF lung. Although this review discusses the interactions of *P. aeruginosa* with *S. aureus* in CF lung, it is noteworthy to mention that *P. aeruginosa* with *S. aureus* also co-exist in the peritoneum of dialysis patients’, diabetic foot wounds, catheters, and in the wounds resulting from skin injury or skin burn. The interactions between these pathogens in the extra-pulmonary locations may involve different mechanisms as the host environment would be completely different from the CF lungs and require further investigations.

## Author Contributions

LB and FG wrote the manuscript and conceived the figures. LB and FG reviewed the manuscript draft. All authors contributed to the article and approved the submitted version. 

## Funding

This work was supported by funding from the Deutsche Forschungsgemeinschaft the Germany’s Excellence Strategy –EXC 2124 – 390838134 ‘Controlling Microbes to Fight Infections’. We acknowledge support by Open Access Publishing Fund of University of Tübingen, Germany.

## Conflict of Interest

The authors declare that the research was conducted in the absence of any commercial or financial relationships that could be construed as a potential conflict of interest.

## Publisher’s Note

All claims expressed in this article are solely those of the authors and do not necessarily represent those of their affiliated organizations, or those of the publisher, the editors and the reviewers. Any product that may be evaluated in this article, or claim that may be made by its manufacturer, is not guaranteed or endorsed by the publisher.

## Abbreviations

AHL, *N*-acylhomoserine lactone; AQNOs, alkyl-hydroxyquinoline N-oxides; CF, cystic fibrosis; CFTR, transmembrane conductance regulator; ETC, electron transport chain; HBD, human β-defensin; HCN, hydrogen cyanide; HQNO, 2-heptyl-4-quinolone N-oxide; PYO, pyocyanin; PQS, *Pseudomonas* quinolone signal; ROS, reactive oxygen species; SCV, small colony variant; ASL, airway surface liquid.
